# Platelet-Rich Plasma in Veterinary Orthopedic Surgery: A Systematic Review and Quality Evaluation on Liquid- and Gel-Based Therapies in Dogs

**DOI:** 10.3390/gels11120994

**Published:** 2025-12-10

**Authors:** Francisco Vidal-Negreira, Mario García-González, Victoria Valiño-Cultelli, Antonio González-Cantalapiedra

**Affiliations:** 1 Rof-Codina Veterinary Teaching Hospital, Faculty of Veterinary, Universidad de Santiago de Compostela, 27002 Lugo, Spain; victoria.cultelli@usc.es; 2 Department of Anatomy, Animal Production and Veterinary Clinical Sciences, Veterinary Faculty, Universidad de Santiago de Compostela, 27002 Lugo, Spain; mariog.gonzalez@usc.es; 3 3B’s Research Group, I3Bs—Research Institute on Biomaterials, Biodegradables and Biomimetics, University of Minho, Headquarters of the European Institute of Excellence on Tissue Engineering and Regenerative Medicine, Barco, 4805-017 Guimarães, Portugal; 4 ICVS/3B’s—PT Government Associate Laboratory, Barco, 4805-017 Guimarães, Portugal

**Keywords:** dog, platelet-rich plasma, gel PRP, veterinary orthopedic surgery, veterinary regenerative medicine, systematic review

## Abstract

The clinical use of platelet-rich plasma (PRP) has gained increasing attention as a regenerative strategy in veterinary orthopedic surgery, yet its efficacy beyond osteoarthritis remains unclear. This systematic review aimed to evaluate the therapeutic potential of liquid and gel PRP formulations as adjuncts in canine orthopedic procedures and to assess the methodological quality of the available evidence. A comprehensive search of PubMed, Scopus, and Web of Science was conducted following PRISMA guidelines. Fourteen eligible studies (six experimental in vivo and eight clinical investigations) including in vivo and clinical investigations of fracture and osteotomy repair and tendon or ligament reconstruction were critically analyzed. Overall, PRP demonstrated safety and biological activity, with early-phase improvements in tissue regeneration and inflammatory modulation; however, long-term functional outcomes were often similar to controls. Gel PRP showed practical advantages in handling, local retention, and the sustained release of growth factors, acting as a transient fibrin scaffold. Quality and risk-of-bias assessments following ARRIVE 2.0, CONSORT, and RoB 2.0 guidelines revealed moderate methodological rigor, with frequent omissions in blinding, sample-size calculation, and preregistration. However, the marked heterogeneity in PRP preparations and outcomes across studies, together with weak evidence for consistent long-term benefits, limits the strength of these conclusions. These findings highlight PRP, particularly in gel form, as a promising biological adjuvant for orthopedic repair in dogs, while emphasizing the need for standardized preparation protocols and harmonized outcome measures to advance its translational application.

## 1. Introduction

Platelet-rich plasma (PRP) is an autologous platelet concentration obtained by centrifugation of whole blood present in a small volume of plasma [1]. It can be administered in liquid form through injection or infiltration for the treatment of diverse medical and surgical conditions such as ligament, joint, or osseus disorders [2]. PRPs are enriched with various factors essential for tissue repair and regeneration like the vascular endothelial growth factor (VEGF), enhancing angiogenesis being fundamental in bone regeneration [3], platelet-derived growth factor (PDGF), transforming growth factor (TGF-b1), or the insulin-like growth factor (IGF) [4,5]. PRP can also be transformed into a gel after the induction of clot formation or gelatinous material. The most commonly used gel activators are chitosan [6], bovine thrombin [7], autologous thrombin [8], calcium chloride [6], or their combinations. By this activation, platelet degranulation and the release of growth factors (GFs), chemokines, and other bioactive metabolites takes place while the clotting of the starting PRP is being performed. The liberation of GFs promotes regenerative processes which include the differentiation and proliferation of angiogenesis and epithelial regeneration [9,10]. Mechanistically, liquid PRP corresponds to a non-activated suspension of platelets in plasma that is injected into the target tissue, where platelets are subsequently activated by endogenous thrombin and tissue factors, leading to in situ fibrin formation and growth-factor release [11]. In contrast, PRP gel is generated ex vivo by adding activators that rapidly convert fibrinogen into a cross-linked fibrin network and trigger platelet degranulation, producing a cohesive fibrin clot that behaves as a transient scaffold, retains platelets and growth factors at the application site, and supports a slower and more localized release as the gel is remodeled [12]. The gel PRP form also has advantages in applicability, as it has been demonstrated that PRP gel presents more adhesiveness, facilitating its application and increasing the time that the formulation stays in the designated space [13,14] while liberating these GFs slowly [15].

Compared with liquid PRP injected into a joint or around soft tissues, gel formulations behave as a transient autologous scaffold that can be molded, adhere to bone and implant surfaces, and remain at the surgical site despite bleeding or joint fluid [16]. This makes PRP gel particularly attractive in orthopedic surgery, where it can be placed directly within fracture or osteotomy gaps, in contact with implants, or combined with three-dimensional scaffolds, potentially enhancing local and sustained growth-factor release and early tissue organization [17]. Clinical studies in dogs have already explored PRP/PRGF gels in fracture lines, high tibial osteotomies, and TTA/MMT-associated bone defects, as well as within composite scaffolds, with mixed but biologically promising effects on radiographic healing and clinical recovery [16,18]

Despite these positive features of PRPs, their efficacy in bone and soft tissue healing is still controversial in human and veterinary medicine [19,20]. The differences in platelet concentration, protocol of elaboration, the condition in which it is used, its route of administration, or the animal model/patient are possible explanations of this variability in the positive effect of PRP in bone or soft tissue healing [21,22].

In veterinary orthopedics, PRPs have been investigated for a broad spectrum of musculoskeletal applications, ranging from bone defects and ligament injuries to joint disorders and tendinopathies [23,24,25], especially in osteoarthritis in dogs, and also with inconsistent results attributable to the same reasons mentioned above [26,27,28,29,30,31,32].

Among these conditions, cranial cruciate ligament rupture (CCLR) is the orthopedic condition mainly diagnosed in dogs leading to lameness, pain, and the instability of the stifle [33,34,35]. This disease severely impacts the animal’s quality of life and mobility, often requiring surgical intervention to restore stability [35,36,37]. The most commonly practiced techniques are proximal tibial osteotomies, either as the tibial plateau leveling osteotomy (TPLO) [38] or by the modified Maquet technique (MMT) [39]. The search for new techniques and resolutions to this condition is still active in the scientific and clinical fields, including the use of PRPs [40].

In this context, and despite the increasing clinical use of PRPs in veterinary orthopedics, the available evidence remains heterogeneous and inconclusive, largely due to variations in preparation methods (leukocyte content, activators, and platelet concentration), administration routes, and timing, as well as the lack of standardized outcome measures [20]. Moreover, most reviews and trials have focused on osteoarthritis, whereas the effects of PRPs on bone consolidation and soft tissue repair remain less clearly defined. Also, the biological behavior and regenerative performance of these PRP gel formulations under experimental and clinical conditions are still poorly understood. Therefore, a systematic and critical evaluation specifically addressing orthopedic models and procedures beyond osteoarthritis is warranted to clarify the true regenerative potential of PRP, particularly in its gel form, and to guide its rational translation into clinical veterinary practice.

Accordingly, this review seeks to determine the biological performance, therapeutic efficacy, and safety of PRP applications in veterinary orthopedic contexts beyond osteoarthritis in dogs. In addition, the study aims to assess the methodological quality and reproducibility of the included works using the ARRIVE 2.0, CONSORT, and RoB 2.0 risk-of-bias tools, identifying common shortcomings and highlighting the need for standardized preparation, characterization, and outcome reporting. Ultimately, this review intends to provide an integrated understanding of PRP, particularly in its gel form, as a translational regenerative biomaterial for future veterinary orthopedic applications.

## 2. Results and Discussion

The literature search initially identified 373 records from electronic databases (PubMed, Scopus, and Web of Science). After removing 144 duplicates, a total of 229 titles and abstracts were screened for eligibility. Of these, 199 studies were excluded because they were unrelated to orthopedic surgery, performed in species other than dogs, were reviews, or did not involve PRP application.

The remaining 30 full-text articles were assessed for eligibility. Among them, 16 studies were excluded after full-text analysis for not meeting the inclusion criteria, as reflected in Table 1.

**Figure 1:**
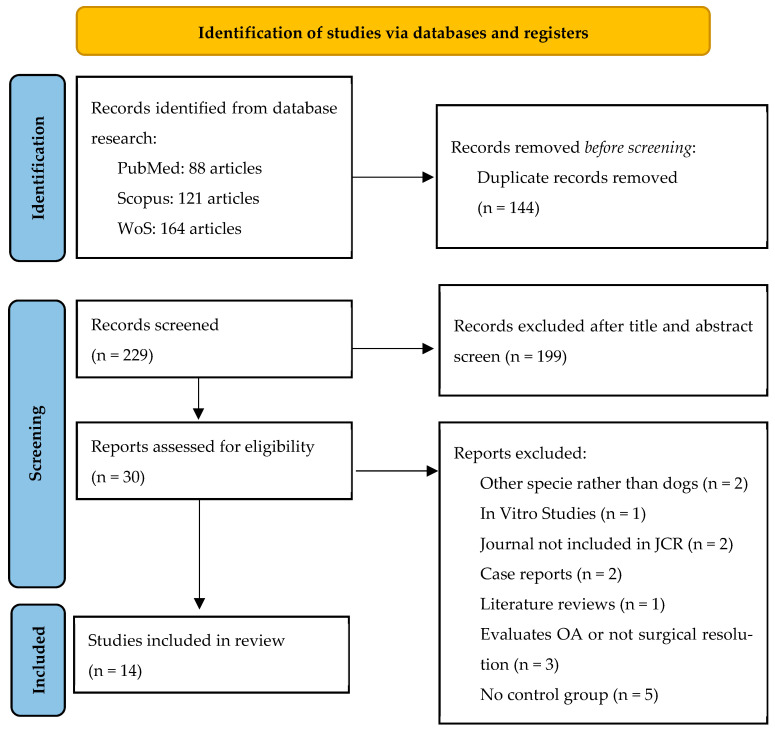
ARRIVE 2.0 flowchart.

Finally, 14 studies met the inclusion criteria and were included in the study. The Cohen kappa coefficient for quality evaluation was 0.851 when using the ARRIVE 2.0 guidelines and was 0.895 when using the CONSORT guidelines. The selection procedure is summarized in Figure 1.

Among the 14 studies included, 7 (50.0%) [16,53,54,55,56,57,58] were clinical prospective studies, 1 (7.1%) [59] was a clinical retrospective study, and 6 (42.9%) [18,60,61,62,63,64] were in vivo studies. A total of 555 dogs were evaluated, corresponding to 656 treated limbs. Among these, 274 limbs (41.8%) received PRP treatment, either in liquid or gel form, while the remainder served as controls or were treated with other procedures. Regarding sex, 316 dogs were male (56.9%) and 197 were female (35.5%). In two studies [60,61] the sex was not reported (39 dogs), and these were included in the total sample size and classified as “sex not reported” in our descriptive summary. The mean age in the studies reported was 5.54 years ± 2.1 years. In five studies [18,53,60,63,64] the age was not specified, only reporting that adult dogs were involved. Weight was represented as kg with a mean of 22.4 ± 9.5 kg. One study [53] only reported percentages.

Most studies reported no loss of animals during follow-up. Drop-outs were recorded in five studies [53,54,56,57,58], with values ranging from 2 to 18 dogs, generally due to postoperative complications or loss of clinical follow-up. In total, 44 dogs were lost, representing an overall attrition rate of 7.9%. Detailed information can be found in Table 2.

### 2.1. Intervention Protocol and Outcome Assessment

The application site and frequency of PRP administration varied among the included studies. The most frequent approach was intra-articular injection, reported in 8 of the 14 studies (57.1%) [55,56,57,58,59,60,61,62], either as a single administration or in repeated doses at 1, 2, 3, 6, and 8 weeks postoperatively.

In seven studies (50.0%) [16,18,53,54,57,58,59], PRP was applied directly at the osteotomy or fracture site, either subcutaneously or within the defect gap, particularly in models of high tibial osteotomies for the resolution of CCLR or bone defect repair. In three protocols (21.4%) [57,58,59], intra-articular administration was combined with application at the osteotomy site to enhance local regeneration.

Two in vivo studies (14.3%) [63,64] described injection into the ligament tunnels, especially in cranial cruciate ligament reconstruction models, while other studies (7.1%) [62] combined the intra-articular and tunnel applications of PRP.

The volume of PRP administered ranged from 1 to 4.9 mL, with a mean of 1.81 ± 1 mL. Only one study reported a markedly higher dose (4.9 mL), corresponding to the direct application of PRP gel into the tibial osteotomy defect in large-breed dogs undergoing TPLO [54].

The follow-up periods reported across the 14 studies ranged from 24 h to 12 months. The shortest follow-up corresponded to immediate postoperative evaluations at 24 h [60], while the longest reached one year [56] after treatment.

A total of three studies (21.4%) [18,55,62] performed follow-ups of ≤2 months, usually at 7, 14, 21, 28, 35, 45, and 60 days, mainly in experimental models assessing early bone or soft tissue healing. One study (7.1%) [59] included intermediate follow-ups between 8 and 10 weeks, often combined with an early evaluation at 10–14 days. Eight studies (57.1%) [16,53,57,58,60,61,63,64] extended the monitoring up to 6–7 months, and one study (7.1%) [56] maintained a long-term follow-up of up to 12 months.

The 14 included studies investigated a variety of orthopedic conditions, predominantly affecting the stifle joint. Six studies (42.9%) [55,60,61,62,63,64] addressed cranial cruciate ligament rupture or related conditions, including partial ligament tears, meniscectomy, reconstruction with autologous tendon grafts, and cases associated with patellar luxation. Three studies (21.4%) [54,56,59] evaluated TPLO procedures, while another three studies (21.4%) [16,18,53] focused on fracture repair, including minimally invasive plate osteosynthesis (MIPO), external fixation, or radial ostectomy models. Two studies (14.3%) [57,58] analyzed MMT.

In terms of the anatomical region, the stifle joint was the most frequently investigated site, appearing in 11 studies (78.6%) [54,55,56,57,58,59,60,61,62,63,64], followed by the tibia in 2 studies (14.3%) [16,53] and the radius/ulna in 2 studies (14.3%) [16,18], including 1 that involved both the tibia and forearm bones [16]. Detailed items are shown in Table 3.

The anesthetic protocol vas reported in 12 studies [18,53,54,55,56,57,58,59,60,62,63,64] while 2 [16,61] did not report it or only stated that “general anesthesia” was used without naming specific drugs, concentrations, and administration routes. The most widely used combination as a preanesthetic or sedation protocol were some a2 agonists like dexmedetomidine [54,55,56,60], xylazine [62], or medetomidine [57,58] with an opioid like hydromorphone [54,56,59], morphine [18,53,57,58,60], or butorphanol [55]. For induction, the most widely used drug was propofol [18,53,54,56,57,58,59,60], and for the maintenance of general anesthesia, the most used inhalation drugs were isoflurane [18,53,54,56,59] and sevoflurane [57,58]. Detailed information about anesthetic protocols is described in Table 4.

The use of scales to asses the functionality of the affected limb was reported in 10 studies [16,18,53,55,57,58,59,60,61,62] while the other 4 studies did not report it or the functionality of the limb was not subjected to study [54,56,63,64]. Information regarding the scales used is shown in Table 4.

### 2.2. Complications

Postoperative complications were reported in 7 of the 14 studies (50.0%) [16,53,57,58,59,61,62], while 2 studies (14.3%) [56,60] explicitly stated the absence of complications and 5 studies (35.7%) [18,54,55,63,64] did not provide information regarding adverse events.

A total of 38 dogs were affected by some type of complication, representing a minimum incidence of 6.8% relative to the total sample size (n = 555). This value likely underestimates the true rate, given that several studies did not specify the number of affected animals.

The most frequent complications were related to implants and fixation devices, observed in three studies (21.4%) [53,57,59] and involving screw breakage, intra-articular screw positioning, or pin loosening and removal (n ≥ 11 dogs). Surgical site infection was reported in one study (7.1%) [59], affecting 16 dogs, and constituted the largest single cause of major complications. Mild local inflammatory reactions, such as sterile acute synovitis or transient pain and swelling, occurred in two studies (14.3%) [61,62], while gastrointestinal signs (gastroenteritis) were described in one case series (n = 4 dogs) [16].

When classified by severity, major complications appeared in one study (7.1%) [59], minor in five (35.7%) [16,58,61,62], and both simultaneously in two (14.3%) [53,57].

Chronologically, three of the events (42.9%) [16,58,59] were late complications, mainly associated with implant fatigue or infection, whereas two studies (28.6%) [61,62] described early-onset events such as synovitis or local inflammation, and two studies (28.6%) [53,57] reported both early and late manifestations within the same cohort.

All reported complications resolved without long-term sequelae. Implant-related problems were managed by removal or replacement, while inflammatory or painful reactions subsided with analgesic treatment or lavage, and gastrointestinal disturbances resolved spontaneously. No study reported mortality, loss of limb function, or adverse reactions directly attributable to PRP administration. A summary of the complications can be reviewed in Table 5.

### 2.3. Type of PRP and Associated Materials

The type and formulation of PRP varied among the included studies. Eight studies (57.1%) [16,53,55,56,59,60,61,62] employed liquid PRP, while four (28.6%) [18,54,63,64] used gel formulations, and two studies (14.3%) [57,58] combined both presentations within the same experimental design. The choice of formulation generally depended on the surgical application: liquid PRP was preferred for intra-articular or tendon/ligament injections, whereas gel PRP was typically applied directly onto bone or osteotomy sites after activation with calcium chloride or thrombin. In most fracture and osteotomy models [18,54,57,58], gel formulations were selected to fill the defect gap or coat implant surfaces, where a cohesive clot was needed to remain in place and function as a transient fibrin scaffold. In contrast, liquid PRP was chosen in studies focused on intra-articular modulation or repeated postoperative injections [55,61], where a freely injectable formulation was required to diffuse within the joint space and allow minimally invasive delivery.

In nine studies (64.3%) [18,53,55,59,60,61,62,63,64], PRP was used as a single biological product without additional biomaterials. The remaining five studies (35.7%) [16,54,56,57,58] incorporated adjunctive components to enhance regenerative potential, including bovine thrombin for activation [54], collagen-based matrices [56], and polylactic acid (PLA) scaffolds [57,58].

Regarding the protocols followed, six studies used a commercial kit [54,55,56,59,60,61] while the other eight [16,18,53,57,58,62,63,64] explained or referenced a manual protocol that could be carried out in clinic. The amount of blood obtained for the PRP preparation was reported in all studies, with a mean of 25.4 ± 28.2 mL and a remarkably high volume of 120 mL in one study [54]. Only three studies [54,60,62] did not report the centrifugation time and velocity, and five studies [53,55,57,58,59] did not report the platelet count before and after or the ratio between the whole blood and PRP preparation. In-depth information about the PRP protocols is detailed in Table 6.

Overall, PRP preparation protocols were highly heterogeneous among studies, differing in platelet and leukocyte concentrations, activation agents, and whether PRP was used in liquid or gel form. Therefore, the results are presented descriptively, and no quantitative pooling or direct comparison between specific preparation methods was undertaken.

### 2.4. Quality Assesment in In Vivo Studies

**Figure 2:**
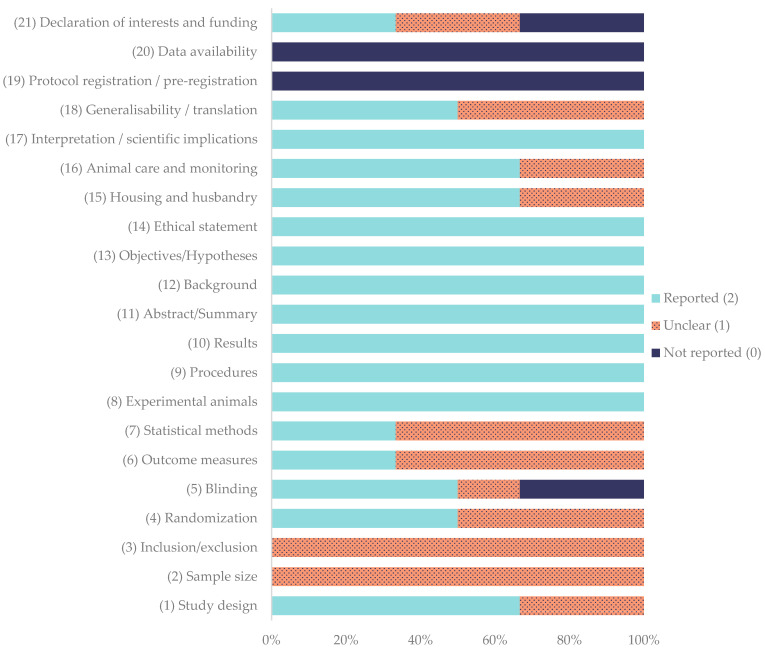
Percentage frequency of each item evaluated using ARRIVE 2.0 guidelines for in vivo studies.

The methodological quality of the included in vivo studies was assessed using the ARRIVE 2.0 guidelines. The corresponding quality coefficients are summarized in Table 7, while the percentage frequency of each ARRIVE item is presented in Figure 2.

Among the evaluated studies, Bozynski et al. (2015) [60], Cook et al. (2016) [61], and Daradka et al. (2025) [62] obtained excellent quality scores, with coefficients ranging between 0.81 and 0.83. Both Souza et al. (2012) [18] and Xie et al. (2013a, b) [63,64] were rated as average, with coefficients of 0.76 and 0.57, respectively. None of the studies achieved the maximum possible coefficient of 1.00.

Regarding individual domains, the study design was adequately reported in 66.7% of the studies [18,60,61,62], while randomization [60,61,62] and blinding [18,60,61] were fully described in 50.0% of the studies; blinding was not reported in 33.3% of the studies [63,64]. Sample size was determined to be unclear in all studies. Outcome measures [60,61] and statistical methods [60,62] reached full reporting in 33.3% of the studies, with the remainder being unclear (66.7%) [18,62,63,64]. In contrast, core descriptive items showed uniformly high adherence: experimental animals, procedures, results, abstracts, backgrounds, objectives, and ethical statements were fully reported in 100% of the studies. Housing and husbandry was totally reported in 66.7% of the studies [18,60,61,62], while this was unclear in 33% of studies [63,64]. Animal care and monitoring was fully reported in 66.7% [18,60,61,62] and not reported in 33.3% [63] of the studies; interpretation/scientific implications were fully reported in 100% of the studies. Generalizability/translation reached 50.0% full reporting [60,61,62]. In contrast, protocol registration and data availability were absent in all studies. Declaration of interests/funding was heterogeneous (reported 33.3% [18,62], unclear 33.3% [60,61], and not reported 33.3% [63,64]).

Quality coefficients ranged from 0.57 to 0.83. None achieved the maximum of 1.00. Collectively, these data indicate good reporting of core methodological descriptors but persistent deficits in sample-size justification, preregistration, data transparency, and, more variably, randomization, blinding, and statistical detail.

### 2.5. Quality Assesment in Clinical Studies

**Figure 3:**
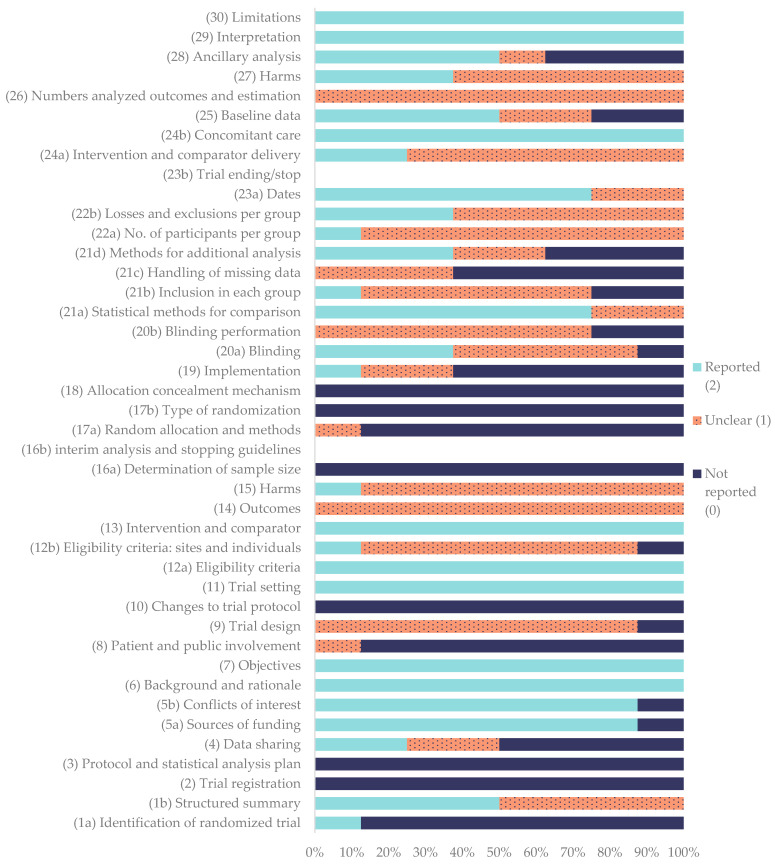
Percentage frequency of each item according to the CONSORT guidelines for clinical studies.

The methodological quality of the included clinical trials was evaluated according to the CONSORT 2025 core checklist [76]. The corresponding quality coefficients for each study are summarized in Table 8, while the percentage frequency of each CONSORT item is illustrated in Figure 3.

Among the assessed publications, Raulinaitė et al. (2023) [55] achieved the highest quality coefficient (0.60), categorized as good. On the other hand, Filgueira et al. (2019) [53] achieved the lowest quality coefficient (0.43), categorized as average. The rest of the studies were categorized as average with coefficients between 0.58 and 0.44. None of the evaluated reports reached a coefficient of 1.00, indicating that no study fully met all the CONSORT criteria.

Regarding the individual CONSORT items, eight were fully reported in all studies (100%): (6) background and rationale and (7) objectives, (11) trial setting, (12 a) eligibility criteria, (13) intervention and comparator, (24b) concomitant care, and (29) interpretation and (30) limitations. On the other hand, several domains, including (2) trial registration, (3) protocol and statistical analysis plan, (10) changes to trial protocol, (16a) sample size determination, (16b) interim analysis and stopping guidelines, (17b) type of randomization, (18) allocation concealment, and (23b) trial ending/stop, were not reported in any of the studies (0%).

The following items were classified as “reported”, that is, the information was adequate and completely provided in most publications: (1a) identification of randomized trial (12.5%), (1b) structured summary (50%), (4) data sharing (25%), (5a) sources of funding (87.5%), (5b) conflicts of interest (87.5%), (20a) blinding (37.5%), (21a) statistical methods for comparison (75%), (23a) dates (75%), and (28) ancillary analyses (50%).

The following items were classified as “unclear”, meaning that the information was incomplete or some sub-elements were missing: (9) trial design (87.5%), (12b) eligibility criteria—sites and individuals (75%), (14) outcomes (100%), (15) harms (87.5%), (17a) random allocation and methods (12.5%), (19) implementation (25%), (20a) blinding (50%) (20b) blinding performance (75%), (21b) inclusion in each group (62.5%), (21c) handling of missing data (37.5%), (21d) methods for additional analysis (25%), (22a) number of participants per group (87.5%), (22b) losses and exclusions per group (62.5%), (24a) intervention and comparator delivery (75%), (25) baseline data (25%), and (26) numbers analyzed for outcomes and estimation.

Finally, the following items were classified as “not reported”, indicating a clear absence of information: (4) data sharing (50%), (8) patient and public involvement (87.5%), (16a) sample size determination (100%), (19) implementation (62.5%), (20b) blinding performance (25%), (21b) inclusion in each group (25%), (21c) handling of missing data (62.5%), and (28) ancillary analysis.

### 2.6. Risk of Bias

**Figure 4:**
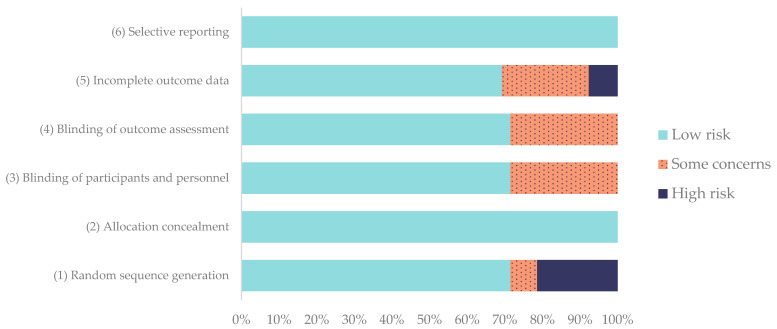
Evaluation of the risk of bias of the studies according to the RoB 2.0 tool for animal studies. Frequencies are expressed in %.

The results of the risk-of-bias assessment are summarized in Figure 4, revealing that the overall risk of bias was satisfactory across the 14 included studies. The Kappa number for the RoB 2.0 assessment was 0.92 between the two evaluators.

Eight of the studies [16,18,57,58,60,62,63,64] were judged as presenting an overall low risk of bias, while the remaining six [53,54,55,56,59,61] presented some concerns, mainly due to insufficient information about randomization or incomplete outcome reporting.

Regarding individual domains, random sequence generation was adequately described in 71.4% of the studies, while allocation concealment was consistently low in risk across all studies (100%). The blinding of participants, personnel, and outcome assessors was performed or clearly reported in 71.4%, although 28.6% had partial or unclear blinding, often in clinical settings where complete masking was not feasible.

Incomplete outcome data showed the greatest variability, with 28.6% of studies presenting high risk or some concerns due to sample loss or unreported follow-up cases. In contrast, selective reporting bias was absent in all studies, as all prespecified outcomes were clearly described (100% low risk).

### 2.7. Discussion

This systematic review provides an updated synthesis of the current evidence regarding the use of platelet-rich plasma (PRP) in canine orthopedic surgery, comparing liquid and gel formulations across both experimental and clinical settings. Overall, the included studies demonstrated that PRP application, regardless of its form, contributes to enhanced bone and soft tissue healing, particularly during the early and intermediate phases of recovery, without increasing the incidence of postoperative complications.

#### 2.7.1. Effects of PRP on the Bone

Evidence from experimental and clinical models demonstrates variable but generally positive effects of PRP on bone healing in dogs. Among the studies reviewed, half (7/14) investigated bone consolidation in tibial osteotomies (TPLO and MMT) or fracture repair models. The type of PRP, its formulation (liquid vs. gel), and the site of application were key determinants of the outcome.

Experimental models have consistently demonstrated that PRP can accelerate early callus formation when applied directly to bone defects. In a controlled radial ostectomy model stabilized with an external fixator, Souza et al. (2012) [18] applied PRP gel activated with calcium chloride within a 2 mm gap and observed faster radiographic and densitometric progression of healing. By day 60, four of the five osteotomies in the PRP group were classified as consolidating compared to only one of the six controls, accompanied by higher bone mineral density and more advanced trabecular organization. Histologically, PRP-treated defects exhibited greater maturity of woven bone and a stronger expression of TGF-β and PDGF, supporting a biological mechanism based on early osteoblast recruitment and matrix deposition [18]. Similar findings were reported by Daradka et al. (2025) [62], using a 10 mm critical-size radial defect filled with PRP in combination with autologous bone graft, where histological and micro-CT analyses at 8 and 12 weeks showed improved vascularization and new bone bridging compared to graft alone [62]. Both studies highlight that local PRP application enhances early osteogenic and angiogenic processes, although the effect appears more pronounced in smaller or graft-supported defects than in isolated critical-size voids.

Clinical data corroborates these preclinical trends, confirming the translational potential of PRP in long-bone fracture repair. López et al. (2019) [16] evaluated dogs with naturally occurring tibial and radial fractures treated with external fixation and intraoperative plasma rich in growth factor (PRGF) infiltration at the fracture site. The PRGF group achieved primary union in all cases, with earlier implant removal than controls and a trend toward faster radiographic consolidation, while mild transient swelling and discomfort were noted at two to four weeks. These results indicate that PRGF accelerates the consolidation phase without compromising safety or function [16]. In contrast, in tibial fractures treated by minimally invasive plate osteosynthesis (MIPO), Filgueira et al. (2019) [53] compared percutaneous injections of PRP, BMC, and chitosan hydrogel. All three treatments supported union, but BMC achieved a significantly shorter healing time, suggesting that although PRP improves biological activity at the fracture site, its osteoinductive potential may be lower than that of cell-rich autologous preparations [53]. Overall, both clinical and experimental studies converge in demonstrating that PRP, especially when activated and delivered directly to the defect, can enhance early bone healing and callus maturation, though its magnitude of effect depends on defect size, the presence of osteoconductive scaffolds, and the biological richness of the comparator treatment.

In canine osteotomies performed for the surgical resolution of cranial cruciate ligament rupture, the reported effects of gel PRP on bone consolidation remain heterogeneous and largely dependent on the formulation and site of application. In a randomized controlled clinical trial, Franklin et al. (2017) [54] evaluated the intraoperative use of a gel PRP activated with calcium chloride and bovine thrombin, placed directly within the TPLO gap of large-breed dogs. Radiographic, ultrasonographic, and magnetic resonance imaging (MRI) analyses performed at 28, 49, and 70 days postoperatively revealed no significant improvements in bone union or callus density compared with the untreated controls. The authors concluded that gel PRP did not accelerate osteotomy healing, with time since surgery and patient age emerging as the only predictors of union rate [54]. Importantly, no increase in adverse events was observed, supporting the safety and local biocompatibility of the PRP formulation used.

In contrast, Aryazand et al. (2023) [59] performed a retrospective study on 110 dogs that underwent TPLO, comparing standard fixation with or without intraoperative treatment using PRP. The product was injected intra-articularly and applied onto the osteotomy plate surface, targeting both the joint environment and periosteal region. Radiographic evaluation at follow-up revealed a significantly higher healing rate in the PRP group (87.6% vs. 51.1%) alongside improved lameness scores and reduced osteoarthritis progression. No differences were detected in infection rates or implant removal, confirming that PRP enhanced bone and soft tissue recovery without compromising surgical safety [59]. This study highlights how low-leukocyte liquid formulations and combined intra-articular/peri-implant delivery may optimize the biological activity of PRP in osteotomy repair.

Further insights come from the MMT assessed by Valiño et al. (2021) [57], which explored the combined effect of a 3D-printed polylactic acid (PLA) wedge scaffold and intraoperative infiltration of PRGF, a standardized platelet concentrate. In this study, PRGF was applied, both in liquid and gel form, within the osteotomy gap and around the surgical site, aiming to enhance the osteointegration of the PLA implant. Over a 12-week follow-up, both the PRGF and control groups achieved adequate ossification and mechanical stability, with no significant differences in bone density or functional outcomes. Interestingly, complete scaffold biodegradation occurred in three PRGF-treated cases, suggesting that the biological activity of PRGF may have accelerated PLA resorption and local tissue remodeling, although without measurable improvement in radiographic consolidation [57].

Collectively, these studies underline that the therapeutic benefit of PRP in osteotomies for CCLR correction is formulation-dependent. While activated PRP gels confined within the osteotomy gap appear to have limited osteogenic impact, liquid preparations applied peri-implant or intra-articularly may achieve superior bone remodeling and joint environment modulation. Nevertheless, the lack of standardized platelet concentration, activation methods, and evaluation criteria among studies continues to hinder direct comparison, emphasizing the need for controlled trials using harmonized protocols to determine the optimal use of PRP in canine TPLO and MMT procedures.

#### 2.7.2. Effects of PRP on Ligament/Tendon Tissue

Experimental studies have provided consistent evidence that PRP promotes early biological remodeling during ligament reconstruction. In two canine ACL reconstruction models, Xie et al. (2013) [63,64] demonstrated that the intra-graft application of CaCl_2_-activated PRP significantly upregulated VEGF and CD31 expression between two and twelve weeks, and increased neurotrophic markers such as NT-3, GAP-43, and NGF [63,64]. These findings indicate that PRP enhances early revascularization and reinnervation of the graft, thereby accelerating the biological maturation phase of ligamentization. Comparable findings have been reported in human ACL reconstruction, where MRI analyses demonstrated that PRP shortened graft-maturation time by nearly half and increased vascular density and cortical bone formation around the tunnels [41,77].

In translational preclinical settings, Bozynski et al. (2015) [60] compared standard NSAID therapy, arthroscopic lavage, and intra-articular injections of leukocyte-reduced PRP for acute CCLR. The PRP-treated dogs exhibited reduced pain, effusion, and lameness, together with improved joint range of motion and a lower severity of ligament pathology on final arthroscopy [60]. Nevertheless, none of the treatments prevented osteoarthritic progression, suggesting that PRP exerts early symptomatic and histologic benefits rather than a clear disease-modifying effect. This aligns with the growing evidence suggesting that PRP provides early symptomatic relief and transient histologic improvements, but lacks robust and consistent disease-modifying properties across long-term clinical outcomes [78].

In naturally occurring partial CCLR, Sample et al. (2018) [56] tested a collagen-PRP hydrogel applied intra-articularly in an attempt to slow synovitis and ligament degeneration over a twelve-month follow-up. The approach proved clinically feasible and biologically active, providing valuable insight into the potential of PRP-based bioaugmentation in partially torn ligaments, although long-term structural protection was not demonstrated.

Clinical data further supports a short-term anti-inflammatory and analgesic effect of PRP formulations. In dogs with unilateral CCLR, Valiño-Cultelli et al. (2022) [58] found no measurable influence of intraoperative PRGF on patellar ligament thickening after MMT.

Taken together, these studies suggest that PRP can enhance the biological environment of the ligament by stimulating vascular and neural remodeling and reducing inflammation, leading to better early function and pain control, although robust long-term evidence of a sustained regenerative or disease-modifying effect is still lacking.

#### 2.7.3. Methodological Quality

The methodological quality of the included studies was average overall, although variability existed across domains.

Experimental in vivo studies showed acceptable adherence to ARRIVE 2.0 recommendations [79] regarding the description of animal characteristics, objectives, and procedural details, but randomization methods, allocation concealment, and statistical planning were often underreported or missing altogether. Sample-size calculations were rarely provided, and none of the studies disclosed protocol registration or data availability statements. The reporting of anesthetic and perioperative analgesic protocols was sparse: only a minority of the studies specified the agents, doses, routes, timing, and monitoring employed. Given that tissue perfusion and nociception influence osteogenesis and pain-sensitive endpoints [80], this under-reporting undermines reproducibility and may confound comparative efficacy signals between PRP formulations. These omissions, while not uncommon in veterinary experimental research, introduce unnecessary variability that may obscure treatment effects and reproducibility in future trials.

Clinical trials demonstrated intermediate compliance with the CONSORT core checklist [76]. Although objectives, eligibility criteria, and surgical procedures were consistently described, fundamental elements such as prospective registration, definition of primary outcomes, and implementation of blinding were generally absent. Few studies detailed how randomization was performed or whether assessors were masked to group allocation, which is particularly problematic for subjective endpoints such as pain or lameness scores. The handling of missing data and reporting of adverse events were also incomplete in most cases. These findings mirror previous evaluations of small animal clinical trials, where design sophistication has increased, but methodological rigor has not advanced at the same pace [81].

Beyond reporting deficiencies, substantial heterogeneity in PRP formulation and application further complicated the assessment of methodological quality. Differences in platelet and leukocyte concentration, activation protocols, final presentation (liquid or gel), dose, and delivery site (intra-articular, intragap, or peri-implant) hindered comparability between studies. Additionally, compositional characterization, such as platelet yield, fibrin architecture, and activation timing, was inconsistently reported, precluding analyses of dose–response relationships or a mechanistic interpretation of outcomes. This variability, combined with the wide range of endpoints used across studies, from radiographic bone union to functional or histological scoring and the absence of quantitative data limited the feasibility of quantitative synthesis impending the elaboration of a meta-analysis in this study.

Despite these limitations, a progressive improvement can be perceived when comparing more recent reports with earlier ones, especially in terms of clearer descriptions of inclusion criteria, treatment protocols, and ethical statements. This trend may reflect a growing awareness among authors, reviewers, and journal editors of the importance of standardized reporting frameworks, as already seen for in vivo experimental studies following the ARRIVE 2.0 guidelines [82]. Nevertheless, the lack of consistent application of CONSORT recommendations continues to hinder the reproducibility and critical appraisal of veterinary clinical trials. Many of the deficiencies identified, particularly incomplete data on randomization, concealment, or blinding, mirror those described in human PRP and orthopedic research, where methodological rigor in pain and functional outcomes is also often limited by the difficulty of implementing masking and by the subjective nature of clinical endpoints [83].

Considering these observations, there is an evident need to harmonize reporting standards across the spectrum of preclinical and clinical veterinary research. While ARRIVE 2.0 provides guidance for experimental in vivo studies and CONSORT focuses on clinical trials, no unified framework currently exists to encompass studies that combine elements of both designs, such as translational research in regenerative medicine. The development of an integrated veterinary reporting guideline, merging key items from ARRIVE 2.0 and CONSORT would facilitate the consistent evaluation of methodological quality and transparency across all stages of the translational continuum. Such harmonization would not only enhance reproducibility and comparability among studies but also strengthen the scientific credibility and clinical applicability of emerging regenerative therapies in veterinary orthopedics.

#### 2.7.4. Risk of Bias

The risk of bias was determined to be low in a general overview of all the studies included. Eight of the studies (57.1%) were judged as having a low overall risk of bias, while the remaining 42.9% presented some concerns, mainly related to incomplete data reporting or insufficient details on randomization procedures. None of the studies were rated as having a high overall risk of bias.

Overall, the risk of bias analysis highlights that randomization and reporting transparency have improved in recent years, reflecting adherence to standardized guidelines such as ARRIVE 2.0, as discussed earlier [82]. However, attrition bias and partial blinding remain important limitations that may influence the interpretation of clinical outcomes, particularly those related to pain and functional recovery, where subjective assessment is common. Similar methodological limitations have been identified in human clinical trials of PRP, where lack of blinding and allocation concealment remain major sources of bias, especially in pain-related outcomes [83]. Future trials should therefore include preregistered protocols, detailed randomization and masking procedures, and complete follow-up datasets to strengthen the reliability and translational value of PRP research in canine orthopedics.

This review is constrained by important sources of clinical and methodological heterogeneity across the included studies. PRP protocols differed in formulation (liquid vs. gel), cellular content (leukocyte-reduced vs. not specified), activation (e.g., CaCl_2_ or bovine thrombin), dose/volume (≈1–4.9 mL), and delivery site (intra-articular, within the osteotomy/fracture gap, or into ligament tunnels). Surgical contexts also varied (TPLO/MMT vs. fracture repair with external fixation or MIPO), and outcomes spanned bone union, ligament/tendon remodeling, functional recovery, and complications, often with different scales and assessment timings. This variability precluded a quantitative synthesis and likely diluted detectable effects when studies were compared qualitatively.

Follow-up schedules were short to intermediate in most studies, limiting inferences about longer-term remodeling, the durability of clinical improvement, or late adverse events. In addition, several studies either did not report complications or reported them incompletely, making our estimated complication rate a minimum rather than a definitive incidence. These reporting gaps matter because they intersect with domains where our risk-of-bias analysis found weaknesses: while allocation concealment and selective reporting were uniformly low risk, incomplete outcome data was the most frequent concern, and partial or unclear blinding was common in clinical settings, factors that can inflate treatment effects for subjective endpoints (e.g., pain and lameness) and bias safety reporting.

The precision of the evidence is further limited by small sample sizes in several trials as by the absence of the calculations used to reach that sample size, retrospective designs on the part of the clinical literature, and co-interventions that are difficult to disentangle (e.g., choice of implant, perioperative NSAIDs, or concurrent biomaterials such as collagen, PLA wedges, or BMC in MIPO). Critically for biologics, many articles did not fully characterize PRP composition (platelet yield, residual leukocytes, fibrin architecture, and activation timing), which hampers dose–response interpretation and prevents the mapping of outcomes to specific product attributes. These deficits directly affect external validity: for example, this study suggests that formulation and target site (gel intragap vs. liquid peri-implant/intra-articular) may influence effect direction, but inconsistent reporting limits firm conclusions.

In this study, PRISMA guidelines were followed, but the evidence base was restricted to English-language, JCR-indexed journals within 2010–2025. This choice increases methodological consistency yet introduces language and indexing bias, and the exclusion of gray literature may favor positive findings. The analysis in this study relied on published data without author queries, so unresolved ambiguities (e.g., exact platelet/leukocyte counts or missing denominators for complications) could not be clarified.

Overall, the signal across studies, particularly for early bone formation in gel intra-defect applications and short-term anti-inflammatory/biologic effects in soft tissues, is biologically plausible and clinically encouraging, but it rests on heterogeneous protocols and moderate risk-of-bias profiles. Future trials should pre-register protocols, report compositional metrics of PRP (platelet and WBC counts, activation method, and final form/viscosity), standardize the timing and site of delivery, and extend follow-up to ≥12 months with blinded outcome assessments.

#### 2.7.5. Clinical Research Implications and Limitations

The overall evidence supports the safety and biological activity of PRP in canine orthopedic surgery, particularly in enhancing early bone and soft tissue healing [22,84]. Liquid or leukocyte-poor formulations applied intra-articularly or around the implant appear more effective than activated gels confined within the osteotomy gap, while local PRP use in fractures and ligament reconstructions promotes early callus formation, angiogenesis, and graft maturation without increasing complications [16,18,63,64]. This pattern largely reflects pragmatic choices in the primary studies: gels were preferentially used when the aim was to retain PRP within a bone gap or ligament tunnel at the time of surgery, whereas liquid preparations were favored for intra-articular injections, repeated dosing, or broader synovial distribution. Nevertheless, the magnitude of benefit remains modest and variable, largely dependent on the formulation, activation, and site of delivery.

From a practical clinical standpoint, a minimally important benefit of PRP in canine orthopedic surgery would be one that either shortens the time to radiographic union sufficiently to modify postoperative management (for example, earlier implant removal or faster return to full weight-bearing) or provides a clear and sustained improvement in objective or validated functional measures, without increasing complications. Several of the included studies suggest such early-phase advantages, for instance, trends toward faster consolidation or earlier improvement in lameness scores, but these effects are often transient, not always accompanied by long-term functional gains, and rarely framed around predefined minimal clinically important differences. As a result, the current evidence supports PRP as a biologically active adjunct with potentially clinically meaningful short-term benefits in selected scenarios, while falling short of definitively demonstrating robust, generalizable improvements across all orthopedic indications.

As viewed in this review, PRP protocols are variable between studies, not allowing for a direct comparison due to significative differences in platelet counts before and after PRP preparation, as stated in the human literature reviews [85]. Future studies should focus on standardizing PRP preparation and reporting, including platelet and leukocyte counts, activation methods, and dosage. Controlled, blinded, and adequately powered clinical trials with objective and long-term outcome measures are still required to define the true therapeutic value of PRP and to distinguish its contribution from that of other biologic adjuvants such as PRGF, bone marrow concentrate, or collagen-based scaffolds.

Among the various formulations, PRP gels deserve consideration as injectable biomaterials that provide both biochemical and structural cues for tissue repair. Their dual nature, delivering growth factors while offering a transient fibrin scaffold, confers advantages over liquid PRP in terms of local retention, sustained release, and spatial stability [86]. Integrating this biomaterial perspective may guide future research toward optimized gel formulations and standardized protocols for clinical use in orthopedic and regenerative veterinary practice.

Another notable limitation identified across the reviewed studies was the inconsistent and often insufficient reporting of anesthetic protocols. Only a minority of authors detailed the use of multimodal anesthesia combining injectable and inhalational agents, local blocks, perioperative analgesia, or prophylactic antibiotics. Inadequate anesthetic or analgesic management can compromise tissue perfusion, a critical factor for osteogenesis [80]. Postoperative pain control was mildly underreported, as only eight studies mentioned the use of analgesics after surgery [16,18,53,54,57,58,59,60]. The absence of appropriate analgesia not only undermines animal welfare but may also induce stress responses capable of altering bone repair outcomes [87]. Evidence supports the use of multimodal regimens that include opioids and anti-inflammatory drugs to effectively minimize nociception and physiological stress [88]. Additionally, it has been proved that the use of NSAIDs does not affect bone regeneration or healing [89], so its use in these studies is more than justified. Furthermore, essential parameters such as heart rate, respiratory rate, and body temperature were rarely monitored, despite being key indicators of anesthetic stability. The lack of standardized anesthetic management and monitoring introduces variability that may bias experimental results and complicate interstudy comparisons. Therefore, future experimental designs should include comprehensive descriptions and justifications of anesthetic and analgesic protocols, adapted to the animal model and the type of bone defect created.

Although most of the included studies assessed limb functionality, the methodologies used were highly heterogeneous, ranging from subjective visual scales, such as the Hudson [65] or Etchepareborde [68] scales, to goniometric measurements [55,61] or force-plate analysis [61]. This lack of standardization limits cross-study comparisons and hinders firm conclusions about the impact of PRP on functional recovery. Overall, the results revealed a clinical improvement or positive trend in animals treated with PRP. This pattern suggests that PRP may contribute to faster recovery or early reduction in pain and inflammation, without necessarily translating into measurable long-term functional gains. Variability in PRP concentration and activation methods, surgical models, the intrinsics of having a subjective method of gait analysis, and evaluation timing likely explains part of this inconsistency. Taken together, the available evidence points to a potential but still inconsistent functional benefit, highlighting the need for future studies to employ standardized and quantitative assessment tools, such as force-plate analysis or kinematic evaluation, to more accurately determine the true clinical efficacy of PRP-based therapies in veterinary medicine.

## 3. Conclusions

This systematic review confirms that platelet-rich plasma (PRP) is a safe and biologically active adjunct in canine orthopedic surgery, showing context-dependent benefits in both bone and soft tissue healing.

Gel formulations, in particular, act as injectable biomaterials that combine the biological potency of platelet-derived growth factors with the structural and temporal support of a transient fibrin matrix, enhancing local retention and the sustained release of bioactive molecules compared with liquid PRP, particularly when molded to fill osteotomy gaps or tendon/ligament defects. This is especially relevant in mechanically dynamic bone and joint environments, where bleeding and synovial fluid can rapidly dilute and wash out liquid preparations.

Experimental and clinical evidence indicates that PRP gels can improve early bone consolidation and modulate the inflammatory environment of soft tissues, especially in osteotomy defects and tendon or ligament reconstruction, suggesting a biologically relevant but variable regenerative effect influenced by differences in formulation, activation, and application protocols.

Standardizing PRP preparation, characterization, and outcome assessment will be essential to define the specific advantages of gel-based systems over liquid preparations and to facilitate their reproducible use as translational regenerative biomaterials in veterinary orthopedics. At the same time, strengthening methodological rigor through improved randomization, blinding, standardized anesthetic protocols, and comprehensive reporting will be fundamental to minimizing bias and ensuring that the observed effects truly reflect the biological efficacy of PRP rather than experimental variability. In this context, future clinical trials should be designed around clearly defined, clinically meaningful benefits, such as a reduction in time to radiographic union that modifies postoperative management, or a sustained improvement in objective gait metrics, so that the true adjunctive role of PRP in canine orthopedic surgery can be more precisely established.

In summary, current evidence does support the routine use of PRP in canine orthopedic surgery beyond osteoarthritis and suggests that PRP, particularly leukocyte-poor liquid formulations, may provide, at best, modest, short-term adjunctive benefits in selected fracture and osteotomy scenarios when combined with appropriate surgical stabilization.

## 4. Materials and Methods

This systematic review was carried out according to the Preferred Reporting Items for Systematic Reviews and Meta-Analyses (PRISMA) [90] statement. The PRISMA checklist can be reviewed in the Supplementary Materials Section.

### 4.1. Search Strategy

The studies have been collected from the following databases: PubMed, Scopus, and Web of Science (WOS). The search was limited to the period of January 2010 to August 2025. The collection of studies was performed in August 2025 by two reviewers (F.V-N and M.G-G).

The search was performed using the following clause: (“platelet-rich plasma” OR PRP) AND (dog OR canine) AND (fracture OR osteotomy OR TPLO OR tendon OR ligament OR arthrodesis OR orthopedic).

### 4.2. PICO Methology

Our study aligned with the PICO methodology, as follows: dogs undergoing orthopedic surgery (P = patients), treated with PRP in liquid or gel form (I = intervention), compared with standard treatments or without PRP application (C = comparison), evaluating outcomes related to bone and soft tissue healing, functional recovery, and postoperative complications (O = outcomes). The PICO question was as follows: What is the effect of platelet-rich plasma in liquid and gel form on bone and soft tissue healing and functional recovery in canine orthopedic surgery as compared to standard treatments or no PRP?

### 4.3. Inclusion and Exclusion Criteria

Studies were included if they met all of the following criteria: (1) in vivo or clinical studies performed in dogs undergoing orthopedic surgical procedures, such as fracture repair, high tibial osteotomies, arthrodesis, or tendon and ligament repair; (2) application of PRP in either liquid or gel form as part of the treatment protocol; (3) presence of a control or comparison group, receiving either a standard treatment or no PRP; (4) assessment of at least one of the following outcomes—bone and soft tissue healing, functional recovery, or postoperative complications; (5) articles written in English and indexed in journals included in the Journal Citation Reports (JCR); and (6) publication date between January 2010 and August 2025.

Studies were excluded if they met any of the following criteria: (1) studies performed in species other than dogs; (2) experimental models or clinical cases focused exclusively on osteoarthritis or other non-surgical applications (e.g., dermatology, ophthalmology, dentistry, and oncology, etc.); (3) in vitro studies, reviews, case reports, conference abstracts, or editorials; and (4) articles lacking sufficient methodological detail or unavailable in full text.

### 4.4. Analysis and Extraction of Parameters of Interest

The studies were evaluated by two evaluators (F. V-N. and M. G-G.) as per the following items: group distribution, number of patients, sex, age, weight, drop-out rate, injection site and frequency, injected volume, and follow-up duration. When demographic data such as sex or age were not reported, these variables were recorded as ‘not reported’. Dogs with missing sex information were included in the overall sample size but were not used for any sex-stratified or adjusted analyses, as only descriptive summaries were performed.

Clinical and surgical information comprised the type of surgery or orthopedic condition treated, the main evaluated outcomes (bone consolidation, soft tissue healing, or functional recovery), the anatomical region involved, anesthetic protocol used, the functionality scale used, and if differences were noted with the functionality within the two groups. Because clinical, radiographic, and functional outcomes were reported using different scoring systems across studies, scores were not mathematically standardized or transformed to a common scale. Instead, we recorded the type of scale used and whether a statistically significant difference between groups was reported and synthesized these findings narratively. When complications were reported, they were classified as major or minor according to Cook et al. (2010) [91], indicating their frequency, period of appearance, and resolution.

Details related to the biological product were also extracted, including the type of PRP (liquid or gel), whether PRP was combined with other substances or biomaterials, how it was prepared, the total volume of blood obtained by venipuncture, the speed and time of centrifugation, and if a platelet count was performed before and after PRP elaboration. Given the expected clinical and methodological heterogeneity, particularly in PRP preparation (centrifugation protocols, platelet and leukocyte concentration, activation method, and final formulation as liquid or gel), a narrative synthesis was performed. For each study, PRP characteristics were extracted and tabulated, and findings were interpreted qualitatively within groups of broadly similar preparations. No meta-analysis or formal comparison between specific PRP protocols was attempted.

Finally, the authors, year of publication, type of article, and study period were also recorded.

### 4.5. Quality Assesment of In Vivo Studies

The methodological quality and reporting transparency of the included in vivo studies were assessed according to the ARRIVE 2.0 guidelines for animal research reporting [79] by two reviewers (F.V-N and M.G-G) independently, and discrepancies were resolved by discussion and consensus. Each of the 21 items was evaluated and scored as “reported” (2 points) when all sub-items were fully described, “unclear” (1 point) when the information was partially provided, and “not reported” (0 points) when the details were absent.

For each study, a quality coefficient was calculated as the sum of the obtained points divided by the maximum possible score (42 points) and then classified as excellent (0.8–1.0), average (0.5–0.8), or poor (<0.5) [92]. In addition, the overall percentages of reported, unclear, and not reported items were calculated for each ARRIVE category.

The ARRIVE 2.0 items were grouped into two categories: the Essential 10 comprising (1) study design, (2) sample size, (3) inclusion and exclusion criteria, (4) randomization, (5) blinding, (6) outcome measures, (7) statistical methods, (8) experimental animals, (9) experimental procedures, and (10) results; and the Recommended Set, including (11) abstract, (12) background, (13) objectives, (14) ethical statement, (15) housing and husbandry, (16) animal care and monitoring, (17) interpretation/scientific implications, (18) generalizability/translation, (19) protocol registration, (20) data access, and (21) declaration of interests.

### 4.6. Quality Assesment of Clinical Studies

The methodological quality and reporting transparency of the included clinical studies were assessed using the CONSORT 2025 core checklist for randomized and non-randomized controlled trials [76]. Each of the 30 core items and their sub-items were independently evaluated and scored as “reported” (2 points) when the information was completely described, “unclear” (1 point) when it was partially provided, and “not reported” (0 points) when the corresponding details were absent. Items that were considered not applicable to a particular study design (such as interim analyses or randomization steps in non-randomized trials) were marked as “–” and excluded from the denominator when calculating item frequencies.

For each clinical study, a quality coefficient was calculated by dividing the sum of the obtained points by the maximum possible score (excluding non-applicable items). The coefficient was then classified as good (≥0.60), average (0.40–0.59), or poor (<0.40), following the interpretative thresholds proposed in previous evaluations of veterinary clinical trials [81]. In addition, the overall proportions of reported, unclear, and not reported items were calculated for each CONSORT item across all included studies.

The 30 items of the CONSORT guidelines were as follows: (1a) identification of a randomized trial, (1b) structured summary, (2) trial registration, (3) protocol and statistical analysis plan, (4) data sharing, (5a) sources of funding, (5b) conflicts of interest, (6) background and rationale, (7) objectives, (8) patient and public involvement, (9) trial design, (10) changes to trial protocol, (11) trial setting, (12a) eligibility criteria, (12b) eligibility criteria for sites and individuals delivering the interventions, (13) intervention and comparator, (14) outcomes, (15) harms, (16a) determination of sample size, (16b) interim analysis and stopping guidelines, (17a) who generated the random allocation and the method used, (17b) type of randomization, (18) allocation concealment mechanism, (19) implementation, (20a) blinding, (20b) how blinding was performed, (21a) statistical methods for comparison, (21b) who is included in each analysis, (21c) handling of missing data, (21d) methods for additional analysis, (22a) number of participants per group, (22b) losses and exclusions for each group, (23a) dates, (23b) trial ending/stop, (24a) intervention and comparator delivery, (24b) concomitant care, (25) baseline data, (26) numbers analyzed for outcomes and estimation, (27) harms, (28) ancillary analysis, (29) interpretation, and (30) limitations.

Two reviewers (F.V-N and M.G-G) independently assessed all articles, and discrepancies were resolved by discussion and consensus. The results were summarized in a frequency table that included, for each CONSORT item, the number of studies in which it was reported, unclear, or not reported, as well as the total number of applicable studies. These frequencies were subsequently used to construct descriptive bar charts illustrating the distribution of reporting completeness across the checklist items.

### 4.7. Risk-of-Bias Assesment

The methodological quality and risk of bias of the included studies were evaluated using the Cochrane Risk of Bias 2.0 (RoB 2.0) tool [93] by two reviewers (F.V-N and M.G-G) independently, and discrepancies were resolved by discussion and consensus. This instrument assesses five domains of potential bias: (1) randomization process, (2) deviations from intended interventions, (3) missing outcome data, (4) measurement of the outcome, and (5) selection of the reported results.

Each domain was judged as presenting a low risk of bias, some concerns, or high risk of bias, following the guidance provided by the RoB 2.0 manual [93]. A general risk of bias for each study was then determined based on the individual domain ratings.

### 4.8. Statistical Analysis

A descriptive analysis was performed, expressing quantitative variables as mean ± standard deviation or median (range) when applicable. Inter-reviewer agreement for the quality and risk-of-bias assessment was quantified using Cohen’s Kappa (κ) statistic. All statistical analyses and graphical representations were performed using the R statistical software (version 4.3.2; R Core Team, Vienna, Austria).

In addition to the demographic, clinical, and outcome variables, PRP preparation characteristics (type of PRP, activation method, blood volume processed, centrifugation protocol, and platelet/leukocyte counts when available) were also summarized descriptively using frequencies and percentages or mean ± standard deviation/median (range).

## Figures and Tables

**Table 1:** Studies excluded after not meeting the inclusion criteria.

Main Reason for Exclusion	No.	References
Species other than dogs	2	[41,42]
In vitro studies	1	[43]
Journal not included in JCR	2	[44,45]
Case reports	2	[46,47]
Literature reviews	1	[2]
Evaluates OA or other non-surgical applications	3	[48,49,50]
No control group	5	[23,24,25,51,52]

OA: Osteoarthritis and No.: number of studies.

**Table 2:** General information on the studies included in the analysis.

Author/Year	Type of Study	Study Period	Treatment Groups		No. of Patient	Sex	Age	Weight (kg)	Drop Out
Aryazand et al., 2023 [59]	Retrospective	Jan. 2018–Dec. 2020	PRP		54	47 neutered males 2 intact males 60 neutered females 1 intact female	6 (1–13) years	29.7	0
			Control		56			28.3	
Bozynsky et al., 2015 [60]	In Vivo	NA	S.O.C.	Control	3	NR	Adult	29.0 (19.7–34)	0
				E. ACL	3				
				Partial tear	3				
			Washout	Control	3				
				E. ACL	3				
				Partial tear	3				
			PRP	Control	3				
				E. ACL	3				
				Partial tear	3				
Cook et al., 2016 [61]	In Vivo	NA	PRP		6	NR	2–5 years	20–27	NR
			Control		6				
Daradka et al., 2025 [62]	In Vivo	Jul. 2019–Jan. 2020	PRP		12	Male	11.4 years	18	0
			Control		12				
Filgueira et al., 2019 [53]	Prospective	2012–2015	PRP		8	13 males 17 females	PRP: 75% > 12 months”	“47% 1–10 kg 26.5% 11–20 kg 26.5% 21–50 kg”	2
			BMC		8		40% < 12 months		
			Chitosan		8				
			Control		8				
Franklin et al., 2017 [54]	Prospective	2017	PRP		27	12 neutered males 15 intact females	4.9 ± 1.7 years	32.2 ± 5.4	4
			Control		33	15 neutered males 17 neutered females 1 intact female	5.7 ± 2.4 years	31.9 ± 5.4	
López et al., 2019 [16]	Prospective	López 2017–2018	PRP		20	11 males 9 females	40.85 months	16.27	0
			Control		23	10 males 13 females	57.17 months	13.07	
Raulinaite et al., 2023 [55]	Prospective	2022–2023	PRP		11	13 males 11 females	2.7 ± 1.1 years	18.2 ±7.5	0
			Control		11				
Sample et al., 2018 [56]	Prospective	Apr. 2013–Jul. 2014	PRP		29	12 neutered males 3 intact males 14 neutered females	5.5 ± 0.5 years (1.6–9.9)	37.1 ± 1.7 (24.1–59.9)	0
			Control		29				
Souza et al., 2012 [18]	In Vivo	NA	PRP		10	4 neutered males 6 neutered females	Adult	4–6 kg	0
			Control		11	4 neutered males 7 neutered females			
Valiño-Cultelli et al., 2021 [57]	Prospective	Dec. 2017–Jul. 2020	PRP		29	16 intact males 4 neutered males 25 intact females 8 neutered females	75.1 ± 45.63 months	26.27 ± 7.41	18
			Control		24		70.16 ± 34.18 months	30.13 ± 13.82	
Valiño-Cultelli et al., 2022 [58]	Prospective	Dec. 2017–Jul. 2020	PRP		29	16 intact males 4 neutered males 25 intact females 8 neutered	75.1 ± 45.63 months	26.27 ± 7.41	18
			Control		24		70.16 ± 34.18 months	30.13 ± 13.82	
Xie et al., 2013a [63]	In Vivo	NA	Normal control		18	Male	Adult	12.5 ± 1.48	0
			Sham		18				
			PRP control		18				
			PRP		18				
Xie et al., 2013b [64]	In Vivo	NA	Normal control		18	Male	Adult	12.5 ± 1.48	0
			Sham		18				
			PRP control		18				
			PRP		18				

NA: not applicable; NR: not reported; S.O.C.: standard of care; and E.: expose.

**Table 3:** Summary of the use of PRP, follow-ups, and anatomical regions.

Author/Year	Injection Site/Times	V Administered (mL)	Follow-Ups	Disease	Anatomical Region
Aryazand et al., 2023 [59]	IA + osteotomy	2	10–14 days + 6–10 weeks	CCLR–TPLO	Stifle
Bozynsky et al., 2015 [60]	IA	2	24 h + 7 weeks + 7 months	CCLR	Stifle
Cook et al., 2016 [61]	IA/1, 2, 3, 6, and 8 weeks	2	6 months	Partial CCLR + menisquectomy	Stifle
Daradka et al., 2025 [62]	IA + tunnels	1	40 days	CCLR	Stifle
Filgueira et al., 2019 [53]	Fracture site	2	0 + 25 + 30 + 60 + 90 + 120 days	Fracture	Tibia
Franklin et al., 2017 [54]	Osteotomy	4.9	28 + 49 + 70 days	CCLR–TPLO	Stifle
López et al., 2019 [16]	Fracture site	1.5	0 + 7 + 14 + 21 + 28 + 60 + 120 + 180 days	Fracture	Radius/ulna Tibia
Raulinaite et al., 2023 [55]	IA	2	0 + 14 + 28 days	CCLR	Stifle
Sample et al., 2018 [56]	IA	2	10 weeks + 12 months	CCLR–TPLO	Stifle
Souza et al., 2012 [18]	Fracture site	1	14 + 21 + 28 + 35 + 45 + 60 days	Radial ostectomy model	Radius
Valiño-Cultelli et al., 2021 [57]	IA + osteotomy	1.5	0 + 1 + 2 + 5 months	CCLR–MMT	Stifle
Valiño-Cultelli et al., 2022 [58]	IA + osteotomy	1.5	0 + 1 + 2 + 5 months	CCLR–MMT	Stifle
Xie et al., 2013a [63]	Tunnels	1	2 + 6 + 12 weeks	CCLR	Stifle
Xie et al., 2013b [64]	Tunnels	1	2 + 6 + 12 weeks	CCLR	Stifle

V.: volume and IA: intra-articular.

**Table 4:** Detailed information about the anesthetic protocols and functionality recovery assessment.

Author/Year	Anesthetic Protocol				Functional Recovery Scale	Main Findings on Functional Assessment
	Premedication/Sedation	Induction	Maintenance	Post-Operatory		
Aryazand et al., 2023 [59]	Hydromorphone 0.1 mg/kg IV	Propofol 6 mg/kg IV	Isoflurane	AB: Cephazolin 22 mg/kg IV TID 10 days PC: NSAID 10 days Gabapentin 5–10 mg/kg 7 to 14 days	Hudson et al., 2004 [65]	Better with PRP
Bozynsky et al., 2015 [60]	Dexmedetomidine 5–10 mg/kg IV Morphine 0.5 mg/kg IV	Propofol 4–8 mg/kg IV	NR	AB: NR Atipamezole PC: Morphine 0.5 mg IV ×1 Tramadol 24 h	Hudson et al., 2004 [65]	Better but *p* > 0.05
Cook et al., 2016 [61]	General anesthesia (NR)				Goniometer Hudson et al., 2004 [65] Pressure sensing walkaway	Better with PRP
Daradka et al., 2025 [62]	Xylazine 1.1 mg/kg IM Ketamine 15 mg/kg IM Meloxicam 0.2 mg/kg SC SID			AB: Amoxicillin 10 mg/kg IM BID 7 days PC: Meloxicam 0.1 mg/kg PO SID 4 days Tramadol 3 mg/kg PO BID/TID 7 days	Goh et al., 2019 [66]	Better with PRP
Filgueira et al., 2019 [53]	Chlorpromazine 0.3 mg/kg IM Morphine 0.25 mg/kg IM Meloxicam 0.2 mg/kg SC	Propofol 4 mg/kg IV	Isoflurane Epidural: Lidocaine 4 mg/kg + bupivacaine 2 mg/kg + tramadol 0.5 mg/kg	AB: Cephalexin 25 mg/kg PO BID 10 days PC: Meloxicam 0.1 mg/kg PO 5 days Dipyrone 25 mg/kg PO TID 7 days Tramadol 3 mg/kg PO TID 5 days	Scott et al., 2011 [67]	Both similar
Franklin et al., 2017 [54]	Dexmedetomidine 5 mg/kg IM Hydromorphone 0.1 mg/kg	Ketamine 5 mg/kg IV Diazepam 0.25 mg/kg IV Propofol 4 mg/kg in RMN	Isoflurane	AB: NR PC: Carprofen 4.4 mg/kg PO SID 7 days	NR	
López et al., 2019 [16]	NR			AB: NR PC: Morphine 0.2 mg IM QUID 24 h Carprofen 4 mg/kg IV SID 24 h	Own visual scale	Both similar
Raulinaite et al., 2023 [55]	Only mild sedation with dexmedetomidine 5 mg/kg IM + butorphanol 0.4 mg/kg IM			NR	Duerr et al., 2014 [37] Goniometer Own visual scale	Better with PRP
Sample et al., 2018 [56]	Dexmedetomidine 2–4 mg/kg IM Hydromorphone 0.1–0.2 mg/kg IM	Propofol 2–10 mg/kg	Isoflurane	NR	NR	
Souza et al. 2012 [18]	Midazolam 0.2 mg/kg IM Morphine 0.5 mg/kg IM	Propofol 4 mg/kg IV Midazolam 0.2 mg/kg	Isoflurane Brachial plex tap lidocaine + bupivacaine 7 mg/kg	AB: Cephalexin 30 mg/kg PO BID 10 days PC: Brachial plex tap repeated Morphine 0.5 mg/kg SID 12 h Tramadol 4 mg/kg PO TID 5 days Meloxicam 0.2 mg/kg PO SID 3 days	Own visual scale	Both similar
Valiño-Cultelli et al., 2021 [57]	Medetomidine 10 mg/kg IM Morphine 0.3 mg/kg IM Meloxicam 0.2 mg/kg IV	Propofol 2 mg/kg IV M.L.K. CRI 1 mL/kg	Sevoflurane M.L.K. CRI 1 mL/kg/h	AB: Cephazolin 22 mg/kg PO 10 days PC: Meloxicam 0.1 mg/kg PO 5 days	Etchepareborde et al., 2011 [68]	Both similar
Valiño-Cultelli et al., 2022 [58]	Medetomidine 10 mg/kg IM Morphine 0.3 mg/kg IM Meloxicam 0.2 mg/kg IV	Propofol 2 mg/kg IV M.L.K. CRI 1 mL/kg	Sevoflurane M.L.K. CRI 1 mL/kg/h	AB: Cephazolin 22 mg/kg PO 10 days PC: Meloxicam 0.1 mg/kg PO 5 days	Etchepareborde et al., 2011 [68]	Both similar
Xie et al., 2013a [63]	NR	Pentobarbital 30 mg/kg IV	NR	AB: Penicillin 3.2 million U IM SID 3 days Streptomycin 1 g IM SID 3 days PC: NR	NR	
Xie et al., 2013b [64]	NR	Pentobarbital 30 mg/kg IV	NR	AB: Penicillin 3.2 million U IM SID 3 days Streptomycin 1 g IM SID 3 days PC: NR	NR	

IV: Intravenous; IM: intramuscular; NA: not applicable; NR: not reported; M.L.K: morphine, lidocaine, and ketamine; U: units; SID: one time daily; BID: two times daily; TID: three times daily; QUID: four times daily; NSAID: non-steroidal anti-inflammatory drug; and PO: oral administration.

**Table 5:** Summary of complications in the studies analyzed.

Author/Year	Complications	N Affected	Early/Late	Classification	Resolution
Aryazand et al., 2023 [59]	Implant infection	16	Late	Major	Implant removal
Bozynsky et al., 2015 [60]	Absence of complications				
Cook et al., 2016 [61]	Sterile acute synovitis	1	Early	Minor	Wash
Daradka et al., 2025 [62]	Mild pain and inflammation	NR	Early	Minor	Analgesic protocol
Filgueira et al., 2019 [53]	Mild inflammation	1	Early	Minor	Implant change or removal
	Intra-articular screw placement	1			
	Screw break	1	Late	Major	
Franklin et al., 2017 [54]	NR				
López et al., 2019 [16]	Mild gastroenteritis	1	Late	Minor	Spontaneous
	Loosening of pins	3			
Raulinaite et al., 2023 [55]	NR				
Sample et al., 2018 [56]	Absence of complications				
Souza et al., 2012 [18]	NR				
Valiño-Cultelli et al., 2021 [57]	Fracture of the distal cortical of tibial crest without displacement	4	NR	Minor	Strict rest
	Apparition of vesicles in the incision region	1			AB administration
	Tension band wiring rupture with or without tibial crest displacement	4		Major	Wire replacement
	Implant rupture	1			Implant removal
Valiño-Cultelli et al., 2022 [58]	Fracture of the distal cortical of tibial crest without displacement	4	NR	Minor	Strict rest
	Apparition of vesicles in the incision region	1			AB administration
	Tension band wiring rupture with or without tibial crest displacement	4		Major	Wire replacement
	Implant rupture	1			Implant removal
Xie et al., 2013a [63]	NR				
Xie et al., 2013b [64]	NR				

NA: not applicable; NR: not reported; N: number; and AB: antibiotic.

**Table 6:** Detailed information about the PRP protocol for each study evaluated.

Author/Year	PRP Type	Combined	Preparation Protocol	Amount of Blood Used (mL)	Centrifugation		Platelet Count
					Speed	Time (min)	
Aryazand et al., 2023 [59]	Liquid	No	Cross et al., 2015 [69] Smith et al., 2016 [70] Arthrex Incorporations	10–15	1500 rpm	5	NR
Bozynsky et al., 2015 [60]	Liquid	No	Arthrex Incorporations	15	NA		P: 2.4× WB 280:1 P:L
Cook et al., 2016 [61]	Liquid	No	Arthrex Incorporations	15	1500 rpm	5	P: 2.5× WB 295:1 P:L
Daradka et al., 2025 [62]	Liquid	No	Dhurat et al., 2014 [71]	20	NR		1 × 10^6^ adjusted P
Filgueira et al., 2019 [53]	Liquid	Activated with 10% CaCl_2_	Own protocol Double centrifugation	4.5	1200 rpm	10	NA
					1600 rpm	10	
Franklin et al., 2017 [54]	Gel	Bovine Thrombin	Arthrex Incorporations	120	NR		P: 7.4× WH L: 5.45 × 10^3^
López et al., 2019 [16]	Liquid	Activated with 10% CaCl_2_	Anitua et al., 2009 [72]	20	460 g	8	P: 2× WH L: < 0.2 × 10^6^ L/mL
Raulinaite et al., 2023 [55]	Liquid	No	Arthrex Incorporations	15	1500 rpm	5	NA
Sample et al., 2018 [56]	Liquid	Collagen	Murray et al., 2007 [73] SmartPReP	32	100 g	14	P: 6.4 × WH
Souza et al., 2012 [18]	Gel	No	Oyama et al., 2004 [74]	8	160 g	20	P: ≥ 338% WH
Valiño-Cultelli et al., 2021 [57]	Both	PLA	Anitua et al., 2009 [72]	27	460 rfc	8	P: 1.5/2 × WH L < 0.2 × 10^6^
Valiño-Cultelli et al., 2022 [58]	Both	PLA	Anitua et al., 2009 [72]	27	460 rfc	8	P: 1.5/2 × WH L < 0.2 × 10^6^
Xie et al., 2013a [63]	Gel	Activated with CaCl_2_	Landesberg et al., 2000 [75]	20	200 g	10	P: 5.03× WH
Xie et al., 2013b [64]	Gel	Activated with CaCl_2_	Landesberg et al., 2000 [75]	20	200 g	10	P: 5.03× WH

CaCl_2_: Calcium chloride; PLA: polylactic acid; rpm: revolutions per minute; rfc: relative centrifugal force; NA: not applicable; NR: not reported; P: platelets; WB: whole blood; and L: leucocytes.

**Table 7:** Summary of quality coefficients using ARRIVE 2.0 guidelines for in vivo studies.

Author	Year	Coefficient	Quality
Bozynsky et al. [60]	2015	0.83	Excellent
Cook et al. [61]	2016	0.81	Excellent
Daradka et al. [62]	2025	0.81	Excellent
Souza et al. [18]	2012	0.76	Average
Xie et al. [63]	2013a	0.57	Average
Xie et al. [64]	2013b	0.57	Average

**Table 8:** Quality coefficients for each study using CONSORT guidelines for clinical studies.

Author	Year	Coefficient	Quality
Aryazand et al. [59]	2023	0.58	Average
Filgueira et al. [53]	2019	0.43	Average
Franklin et al. [54]	2017	0.56	Average
Lopez et al. [16]	2019	0.46	Average
Raulinaite et al. [55]	2023	0.60	Good
Sample et al. [56]	2018	0.57	Average
Valiño-Cultelli et al. [57]	2021	0.44	Average
Valiño-Cultelli et al. [58]	2022	0.44	Average

## Data Availability

The original contributions presented in this study are included in the article. Further inquiries can be directed to the corresponding authors.

## Supplementary Materials

The following supporting information can be downloaded at https://www.mdpi.com/article/10.3390/gels11120994/s1. Table S1: PRISMA checklist. Table S2: PRISMA Abstract checklist [94].

## Abbreviations

The following abbreviations are used in this manuscript:

ARRIVE	Animal Research: Reporting of In Vivo Experiments
BMC	Bone Marrow Concentrate
CCLR	Cranial Cruciate Ligament Rupture
CONSORT	Consolidated Standards of Reporting Trials
GFs	Growth Factors
IGF	Insulin Growth Factor
JCR	Journal Citation Reports
lPRP	Leukoreduced PRP
MIPO	Minimally Invasive Plate Osteosynthesis
MMT	Modified Maquet Technique
OA	Osteoarthritis
PDGF	Platelet-Derived Growth Factor
PLA	Polylactic Acid
PRGF	Plasma Rich in Growth Factors
PRISMA	Preferred Reporting Items for Systematic Reviews and Meta-Analyses
PRP	Platelet-Rich Plasma
RoB 2.0	Risk of Bias
SYRCLE	Systematic Review Centre for Laboratory Animal Experimentation
TGF- b1	Transforming Growth Factor
TPLO	Tibial Plateau Leveling Osteotomy
VEGF	Vascular Endothelial Growth Factor
WOS	World of Science
